# Admission complete blood count-derived inflammatory indices for risk stratification in hemorrhagic fever with renal syndrome: comparative performance of NLR, PLR, SII, LMR, and the neutrophil-to-red blood cell ratio

**DOI:** 10.3389/fimmu.2026.1863714

**Published:** 2026-06-30

**Authors:** Zhuoran Xiao, Xingchi Chen, Min Wei, Shasha Wu, Shuxiang Zhao, Dandan Suo, Shangying Zhao, Meng Li, Xiaofei Yang, Chao Fan, Jianqi Lian, Chuantao Ye, Jing Zhang

**Affiliations:** 1 Department of Infectious Diseases, Second Affiliated Hospital of The Fourth Military Medical University, Xi’an, Shaanxi, China; 2 Department of Infectious Diseases, Weinan Central Hospital, Weinan, Shaanxi, China

**Keywords:** biomarker, hemorrhagic fever with renal syndrome, LMR, neutrophil-to-red blood cell ratio, NLR, NRR, PLR, SII

## Abstract

**Background:**

Hemorrhagic fever with renal syndrome (HFRS) requires early risk stratification, yet the comparative value and reproducibility of complete blood count (CBC)-derived inflammatory indices remain unclear. We compared admission neutrophil-to-lymphocyte ratio (NLR), platelet-to-lymphocyte ratio (PLR), systemic immune-inflammation index (SII), lymphocyte-to-monocyte ratio (LMR), and neutrophil-to-red blood cell ratio (NRR) for identifying severe disease and 28-day mortality.

**Methods:**

We retrospectively analyzed 342 adults with serologically confirmed HFRS treated at the Second Affiliated Hospital of the Fourth Military Medical University from January 2020 to May 2026. Clinical severity was classified as mild or severe, and prognosis as 28-day survival or non-survival. Group comparisons, receiver operating characteristic (ROC) curves, exploratory Kaplan–Meier analyses, and age- and sex-adjusted single-marker logistic models were used to evaluate CBC-derived indices and conventional laboratory markers. An independent 100-patient cohort from Weinan Central Hospital was analyzed using identical endpoint definitions and a prespecified validation framework.

**Results:**

In the derivation cohort, 327 patients survived and 15 died within 28 days. For severe disease, NRR had the highest AUC among CBC-derived indices (0.750), followed by NLR (0.657), SII (0.467), LMR (0.400), and PLR (0.354). For 28-day mortality, NLR had the highest AUC (0.751), followed by NRR (0.694), LMR (0.558), SII (0.479), and PLR (0.345). After age and sex adjustment, NRR was associated with severe disease and 28-day mortality. In the validation cohort, NRR retained the highest severe-disease AUC (0.701) and remained independently associated with severe disease. For mortality, NRR showed the highest AUC (0.697), but its adjusted association was not significant, and Kaplan–Meier analysis showed no significant separation.

**Conclusions:**

NRR showed promising performance for identifying severe HFRS and was associated with mortality in the derivation cohort, but mortality findings were not confirmed externally. NRR may serve as a candidate CBC-derived adjunctive marker, requiring prospective validation.

## 1 Introduction

Hemorrhagic fever with renal syndrome (HFRS) remains an important rodent-borne zoonosis in East Asia, particularly in China, where Hantaan virus and Seoul virus continue to cause substantial morbidity (1, 2). Clinically, HFRS is characterized by fever, hemorrhagic manifestations, thrombocytopenia, acute kidney injury, and variable degrees of capillary leak, shock, and multi-organ dysfunction (2–4). Endothelial dysfunction and dysregulated innate immune activation are central to pathogenesis, with neutrophil-driven inflammatory injury contributing to barrier disruption and disease amplification (3–8). Because deterioration may occur early and abruptly, practical biomarkers that support admission triage remain clinically relevant.

Routine HFRS evaluation already includes white blood cell count, neutrophils, platelets, renal function, procalcitonin (PCT), and C-reactive protein (CRP), but these variables individually provide only partial insight into the interaction between inflammation, endothelial leakage, thrombocytopenia, and organ injury (9–11). Over the last decade, complete blood count (CBC)-derived composite indices, especially NLR, PLR, SII, and LMR, have been increasingly explored as low-cost inflammatory surrogates across infectious and immune-mediated disorders (12–16). In hantavirus infection, NLR has shown a relationship with acute disease burden (12), yet comparative evidence across multiple CBC-derived indices in HFRS remains limited.

Our group previously identified pentraxin-3 as a useful biomarker in HFRS, but that approach depends on non-routine immunoassays and is less suitable for rapid bedside stratification (17). In contrast, the neutrophil-to-red blood cell ratio (NRR) can be derived directly from a standard CBC. NRR may be biologically plausible in HFRS because it combines neutrophil burden with an RBC-related denominator that may partly reflect the circulatory concentration context accompanying plasma leakage and hemoconcentration (7, 8, 18). However, RBC count is not a direct measure of hemoconcentration and may be affected by anemia, hydration status, sex, altitude, hemorrhage, and fluid therapy. Therefore, the predictive value of NRR for disease severity and prognosis in the early stages of HFRS requires further clarification.

The present study therefore aimed to compare NLR, PLR, SII, LMR, and the less explored NRR in a retrospective HFRS cohort and an independent external validation cohort, to determine whether NRR may serve as a candidate admission marker for clinical severity and short-term mortality.

## 2 Materials and methods

### 2.1 Study design and patient selection

This retrospective observational study included consecutive adult patients diagnosed with HFRS at the Second Affiliated Hospital of the Fourth Military Medical University between January 2020 and May 2026. Eligible patients had (1) a clinical presentation compatible with HFRS, (2) positive hantavirus-specific IgM serology, (3) admission during the acute disease phase, and (4) complete baseline clinical and laboratory data. Exclusion criteria were pregnancy, chronic kidney disease, autoimmune disease or immunodeficiency, active malignancy, documented bacterial coinfection or other major concomitant infection, preadmission systemic glucocorticoid or immunosuppressive therapy, or incomplete data. After applying these eligibility and exclusion criteria, 342 patients were included in the derivation cohort for analysis. An independent external validation cohort comprising 100 HFRS patients from Weinan Central Hospital was selected according to the same prespecified inclusion and exclusion criteria and analyzed separately from the derivation cohort to assess reproducibility and generalizability.

### 2.2 Clinical definitions and endpoints

Clinical severity was categorized according to conventional HFRS criteria as mild, moderate, severe, or critical (19–21). For comparative analyses, mild and moderate cases were grouped as the mild category, whereas severe and critical cases were grouped as severe disease. The prognostic endpoint was 28-day survival status, defined as all-cause mortality within 28 days after admission. Patients who died within 28 days were classified as non-survivors, whereas those alive at day 28 were classified as survivors. This binary survival status was used for logistic regression analyses, and time from admission to death or censoring at day 28 was used for time-to-event analyses.

### 2.3 Laboratory testing and calculation of inflammatory indices

Admission blood counts were obtained using an automated hematology analyzer (Sysmex XN-9000, Sysmex Corporation, Kobe, Japan). These measurements provided white blood cell (WBC), absolute neutrophil, lymphocyte, monocyte, platelet (PLT), and red blood cell (RBC) counts. Cell count units were reported consistently as ×10^9/L for WBC, neutrophils, lymphocytes, monocytes, and platelets, and ×10^12/L for RBC. Biochemistry and inflammatory markers, including creatinine and PCT, were assessed by routine institutional methods.

CBC-derived indices were calculated using the numeric values reported by the analyzer: NLR = neutrophil count/lymphocyte count; PLR = platelet count/lymphocyte count; SII = platelet count × neutrophil count/lymphocyte count; LMR = lymphocyte count/monocyte count; NRR = neutrophil count/RBC count.

### 2.4 Statistical analysis

Continuous variables were non-normally distributed and are summarized as medians with interquartile ranges (IQRs). Categorical variables are presented as counts and percentages. Between-group comparisons were performed using the Mann–Whitney U test for continuous variables and the Pearson χ² test or Fisher’s exact test for categorical variables, as appropriate.

Receiver operating characteristic (ROC) curve analysis was used to evaluate the discriminative performance of complete blood count (CBC)-derived indices and conventional laboratory markers for severe disease and 28-day all-cause mortality. Area under the curve (AUC) values were reported with 95% confidence intervals (CIs). For markers showing an inverse association with disease severity or mortality risk, raw AUCs were interpreted with explicit consideration of marker directionality, and direction-corrected AUCs were examined in sensitivity analyses where appropriate. Bootstrap resampling with 1,000 successful replicates was used to estimate optimism-corrected AUCs in the derivation cohort. Pairwise DeLong tests compared NRR with the other CBC-derived indices using direction-corrected AUCs. An approximate ROC power/precision assessment was performed for severe-disease discrimination, where the event distribution was adequate.

Adjusted associations with severe disease and 28-day mortality were assessed using prespecified single-marker logistic regression models adjusted for age and sex. Each candidate marker was entered into a separate model. No stepwise selection or data-driven multivariable variable selection was performed. This parsimonious modeling strategy was adopted *a priori* to reduce the risk of overfitting, particularly because the 28-day mortality endpoint included only 15 events in the derivation cohort. Odds ratios (ORs) with 95% CIs are presented per raw-unit increase in the main analyses. To improve comparability across biomarkers measured on different scales, per-IQR effect estimates were additionally evaluated as a sensitivity analysis. Events-per-parameter were summarized using the smaller outcome class per three-parameter single-marker model. Variance inflation factors (VIFs) were examined, and Hosmer-Lemeshow tests were used as descriptive checks for gross calibration.

Kaplan-Meier analyses were used as exploratory visualizations of 28-day event-free survival. To avoid preferential optimization of any single marker, the primary Kaplan–Meier analyses used median cutoffs for all five CBC-derived indices. Differences between groups were assessed using the log-rank test, and hazard ratios (HRs) with 95% CIs were estimated as descriptive effect measures. Youden index-derived cutoffs were evaluated only in sensitivity analyses and were not interpreted as validated clinical thresholds. All analyses of 28-day mortality, cutoff-based stratification, and time-to-event outcomes were considered exploratory. A two-sided P value <0.05 was considered statistically significant. The external validation cohort was analyzed separately from the derivation cohort using the same endpoint definitions, biomarker calculations, and prespecified analytical framework.

## 3 Results

### 3.1 Cohort characteristics

A total of 342 patients were included in the cohort after complete-case screening. The median age was 48.00 years (IQR 35.00-57.00), and 267 patients (78.1%) were male. According to the 28-day mortality endpoint, 327 patients survived and 15 patients did not survive. For severity analyses, 159 patients were classified as severe and 183 as mild.

Baseline characteristics according to survival status are summarized in **Table 1**. Age and sex did not differ significantly between survivors and non-survivors. Non-survivors had higher neutrophil counts (13.77 vs 7.71 ×10^9/L, P = 0.001), higher RBC counts (5.35 vs 4.62 ×10^12/L, P = 0.009), lower platelet counts (17.00 vs 50.00 ×10^9/L, P = 0.023), higher WBC counts (18.11 vs 13.04 ×10^9/L, P = 0.019), and higher PCT levels (10.10 vs 2.19, P = 0.009). Creatinine did not differ significantly between groups. Length of stay was shorter among non-survivors (5.00 vs 13.00 days, P = 0.016), consistent with early fatal events in this endpoint definition.

**Table 1:** Baseline characteristics and early clinical course in the derivation cohort according to 28-day survival status.

Characteristic	Survivors (n=327)	Non-survivors (n=15)	P value
Cohort descriptor			
Patients, n	327	15	
Demographics and presentation			
Age, years	48.00 [35.00, 57.00]	48.00 [38.00, 68.00]	0.235
Male sex, n (%)	257 (78.6)	10 (66.7)	0.44
Routine laboratory markers and clinical course			
Neutrophils, ×10^9/L	7.71 [5.00, 11.86]	13.77 [10.64, 31.98]	0.001
Lymphocytes, ×10^9/L	3.35 [1.79, 5.08]	4.09 [1.67, 5.36]	0.6
Monocytes, ×10^9/L	1.11 [0.64, 3.00]	0.96 [0.71, 2.55]	0.853
Red blood cells, ×10^12/L	4.62 [3.99, 5.26]	5.35 [4.97, 6.00]	0.009
Platelets, ×10^9/L	50.00 [28.00, 82.00]	17.00 [13.00, 77.50]	0.023
White blood cells, ×10^9/L	13.04 [8.96, 19.55]	18.11 [15.55, 40.95]	0.019
Procalcitonin	2.19 [0.71, 6.19]	10.10 [2.93, 12.31]	0.009
Creatinine, μmol/L	221.80 [114.35, 362.50]	164.40 [105.50, 273.20]	0.188
Length of stay, days	13.00 [9.00, 17.00]	5.00 [2.00, 15.50]	0.016
CBC-derived inflammatory indices			
NLR	2.82 [1.72, 4.23]	5.08 [3.20, 8.20]	0.001
PLR	14.85 [6.43, 41.07]	4.96 [2.25, 58.26]	0.042
SII	118.59 [62.99, 268.81]	74.10 [58.45, 524.97]	0.782
LMR	2.38 [1.35, 3.89]	3.00 [1.64, 3.45]	0.444
NRR	1.78 [1.14, 2.66]	2.57 [1.72, 5.82]	0.011

### 3.2 Admission CBC-derived inflammatory indices differ in distinct ways by severity and outcome

**Figure 1:**
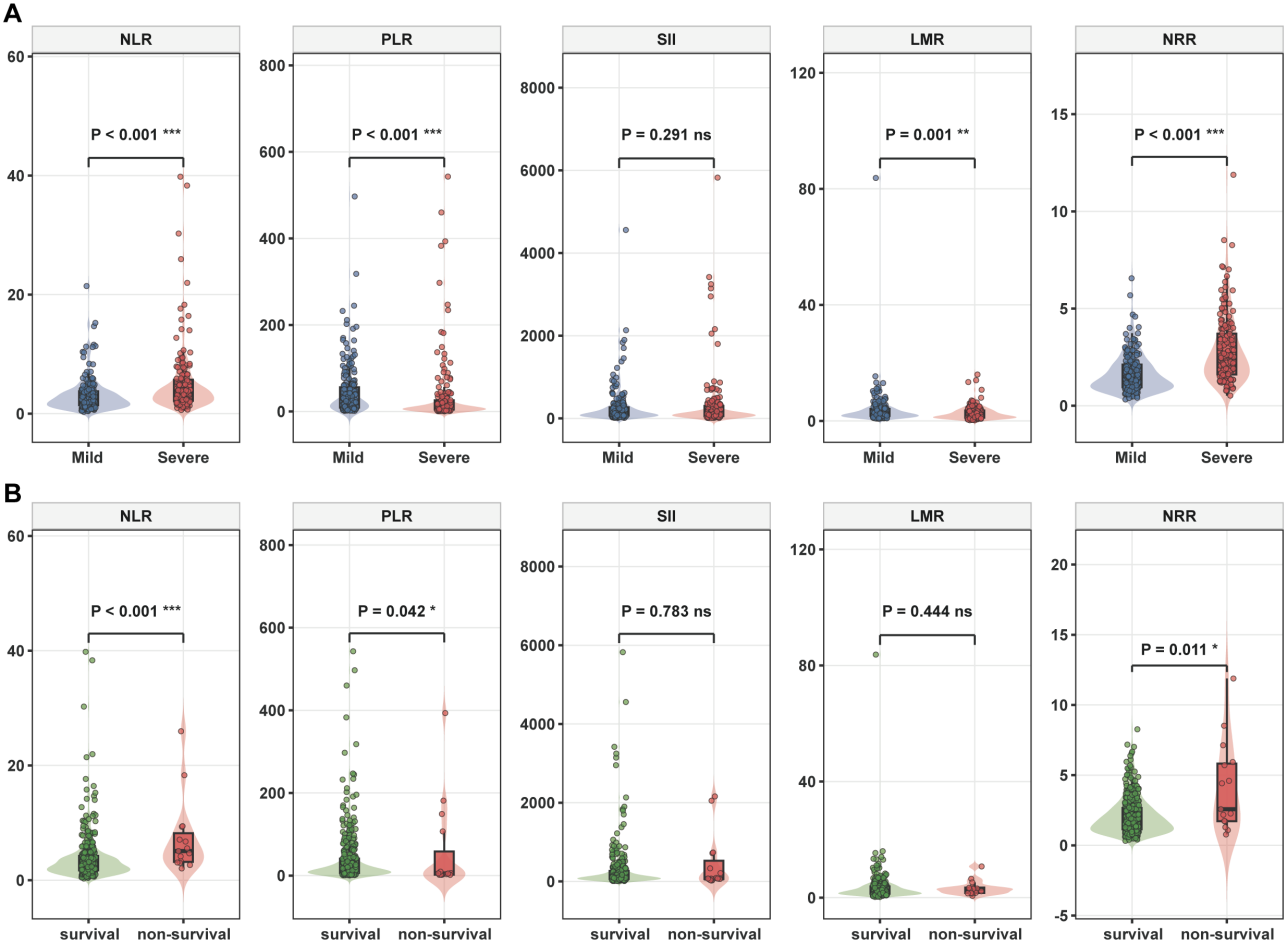
Admission CBC-derived inflammatory indices in the derivation cohort stratified by HFRS severity and 28-day survival status. **(A)** Violin/box plots comparing NLR, PLR, SII, LMR, and NRR between non-severe and severe disease. **(B)** The same five indices compared between survivors and non-survivors within 28 days after admission. *: p < 0.05, **: p < 0.01, ***: p < 0.001, ns: not significant (p > 0.05).

The five CBC-derived indices displayed different patterns across HFRS severity states (**Figure 1A**). NRR and NLR were higher in severe disease (both P < 0.001), whereas PLR was lower in severe disease (P < 0.001). LMR also differed by severity (P = 0.001), with lower values in the severe group. SII did not show significant severity-group separation (P = 0.291).

A related but not identical pattern was observed for 28-day survival status (**Figure 1B**). NLR was significantly higher in non-survivors than in survivors (P < 0.001), and NRR was also higher in non-survivors (P = 0.011). PLR again moved in the inverse direction and was significantly lower among non-survivors (P = 0.042). SII (P = 0.783) and LMR (P = 0.444) were not informative for mortality-group discrimination. These results indicate that PLR carries disease-related information in HFRS, but its lower values indicate higher risk because thrombocytopenia is a disease hallmark.

### 3.3 Conventional laboratory markers in the expanded derivation cohort

**Figure 2:**
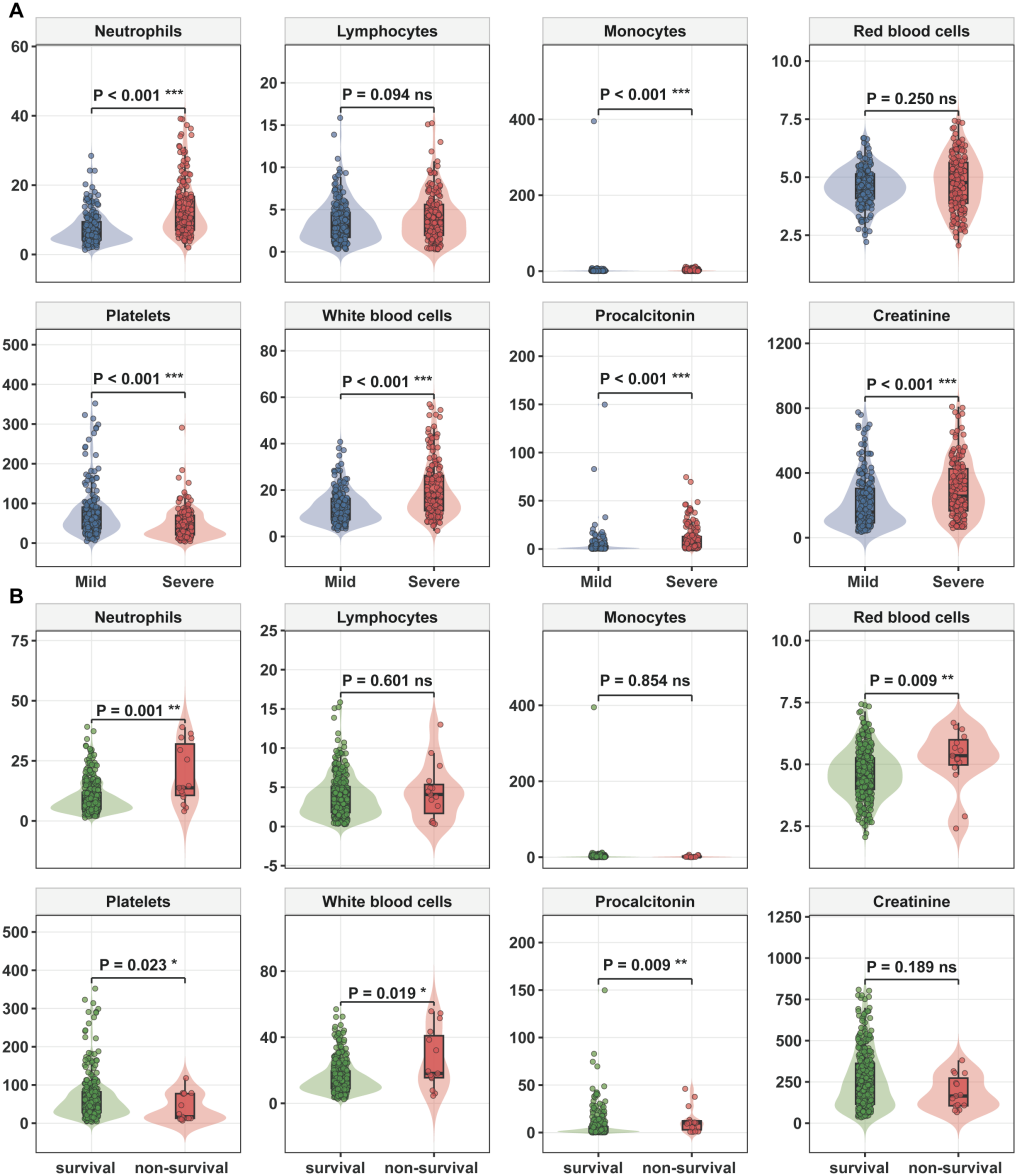
Conventional laboratory markers in the derivation cohort according to HFRS severity and 28-day survival status. **(A)** Neutrophils, lymphocytes, monocytes, RBC, platelets, WBC, PCT, creatinine, and length of stay in non-severe versus severe disease. **(B)** The same variables in survivors versus non-survivors within 28 days after admission. *: p < 0.05, **: p < 0.01, ***: p < 0.001, ns: not significant (p > 0.05).

Conventional laboratory markers also showed distinct severity- and survival-related patterns (**Figure 2**). In the severity comparison, severe cases had higher neutrophil counts (P < 0.001), monocyte counts (P < 0.001), WBC counts (P < 0.001), PCT levels (P < 0.001), and creatinine levels (P < 0.001), as well as lower platelet counts (P < 0.001). Lymphocyte count (P = 0.094) and RBC count (P = 0.250) did not differ significantly by severity in this comparison (**Figure 2A**).

In the 28-day survival comparison, non-survivors had higher neutrophil counts (P = 0.001), RBC counts (P = 0.009), WBC counts (P = 0.019), and PCT levels (P = 0.009), together with lower platelet counts (P = 0.023) (**Figure 2B**). Lymphocytes, monocytes, and creatinine did not differ significantly between survivors and non-survivors. These results support a combined inflammatory and hematologic signal in early fatal HFRS, while also showing that creatinine alone was not a mortality discriminator in the expanded complete-case cohort.

### 3.4 ROC analysis for severe disease and 28-day mortality

**Figure 3:**
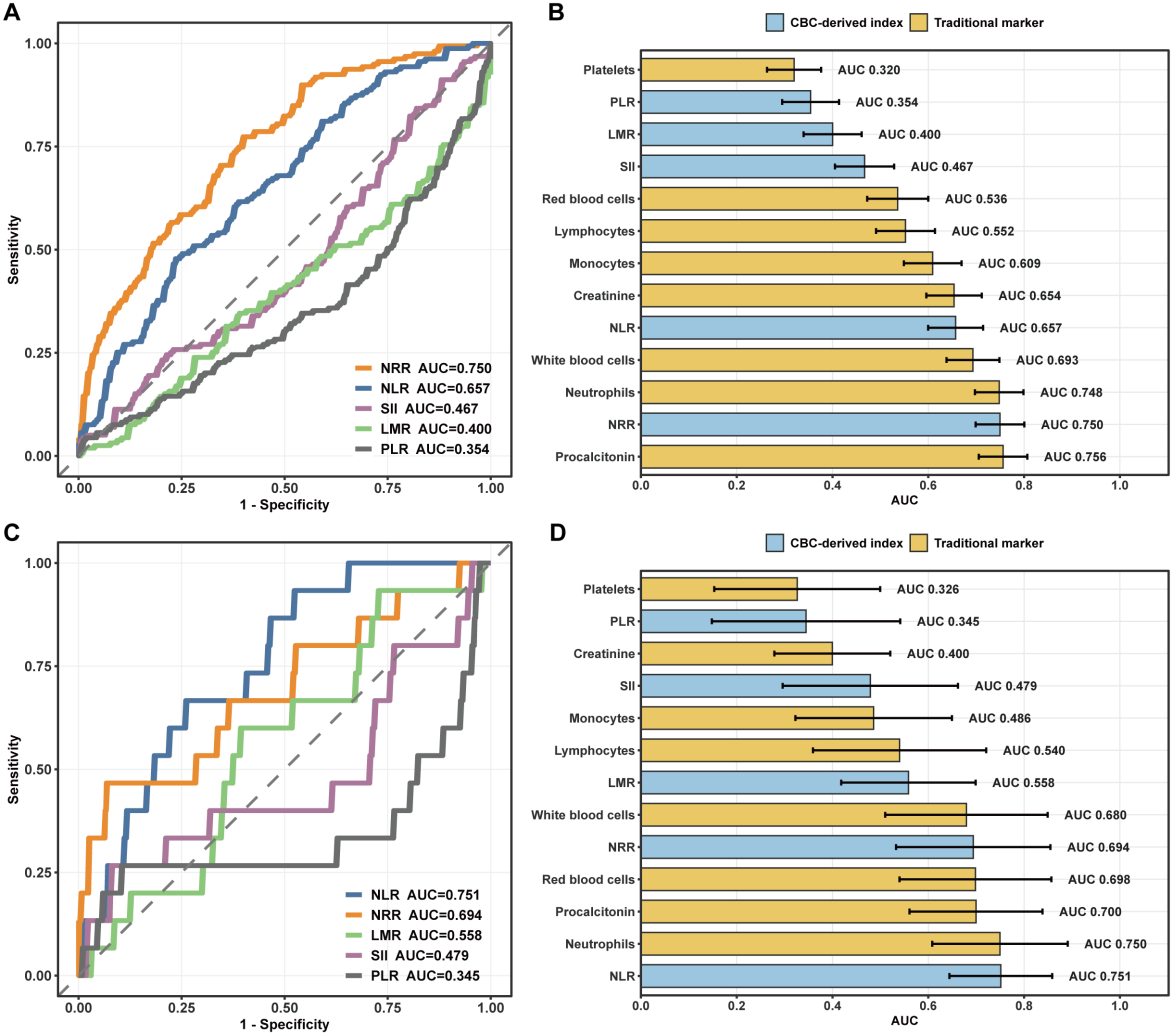
ROC-based comparison of CBC-derived inflammatory indices and routine laboratory markers in the derivation cohort. **(A)** Overlaid ROC curves of the five CBC-derived indices for severe disease. **(B)** AUC summary plot for severe disease. **(C)**Overlaid ROC curves of the five CBC-derived indices for 28-day mortality. **(D)** AUC summary plot for 28-day mortality.

ROC analysis for severe disease showed that NRR had the highest raw AUC among the five CBC-derived indices (AUC 0.750), followed by NLR (0.657), SII (0.467), LMR (0.400), and PLR (0.354) (**Figure 3A**). The AUCs below 0.50 for PLR, LMR, and SII reflect their inverse direction in relation to severe disease rather than absence of any disease-related information. After direction correction, the AUCs were 0.646 for PLR, 0.600 for LMR, and 0.533 for SII. Pairwise DeLong testing of direction-corrected AUCs showed that NRR had a higher AUC than the other CBC-derived indices for severe disease (NRR vs NLR, P = 0.006; vs PLR, P < 0.001; vs LMR, P < 0.001; vs SII, P < 0.001). In bootstrap analysis, the optimism-corrected AUC for NRR was 0.750, similar to the apparent AUC, and the approximate power for the observed severe-disease AUC was >0.99.

When the CBC-derived indices were compared with routine markers for severe disease, PCT (AUC 0.756), NRR (0.750), and neutrophils (0.748) were closely clustered, while WBC (0.693), NLR (0.657), creatinine (0.654), monocytes (0.609), lymphocytes (0.552), RBC (0.536), SII (0.467), LMR (0.400), PLR (0.354), and platelets (0.320) showed lower raw AUCs (**Figure 3B**). These findings support NRR as a promising CBC-derived marker for severe disease, but they do not establish statistical superiority over PCT or absolute neutrophil count.

For 28-day mortality, NLR achieved the highest raw AUC among the five CBC-derived indices (0.751), followed by NRR (0.694), LMR (0.558), SII (0.479), and PLR (0.345) (**Figure 3C**). Among all evaluated markers, NLR (0.751) and neutrophils (0.750) had the highest AUCs, followed by PCT (0.700), RBC (0.698), NRR (0.694), WBC (0.680), LMR (0.558), lymphocytes (0.540), monocytes (0.486), SII (0.479), creatinine (0.400), PLR (0.345), and platelets (0.326) (**Figure 3D**). Therefore, the revised analysis supports NRR as an informative candidate marker, but NLR and absolute neutrophils showed stronger mortality discrimination in the derivation cohort. Direction-corrected AUCs for PLR and SII were 0.655 and 0.521, respectively, reflecting inverse associations. By DeLong testing, NRR did not differ significantly from any other CBC-derived index for 28-day mortality (all P ≥ 0.113; NRR vs NLR, P = 0.572). The optimism-corrected AUC for NRR was 0.690. These findings support cautious, descriptive interpretation of mortality AUC rankings.

### 3.5 Adjusted associations analysis

**Figure 4:**
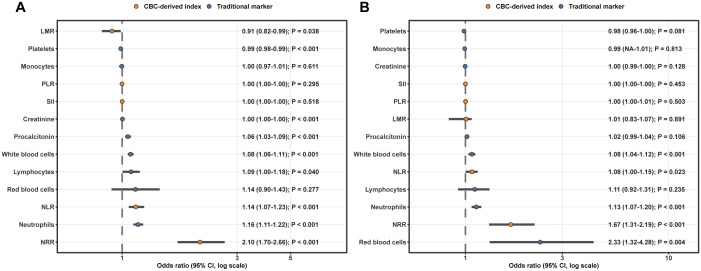
Age- and sex-adjusted associations of candidate markers with severe disease and 28-day mortality in the derivation cohort. **(A)** Forest plot of adjusted odds ratios for severe disease. **(B)** Forest plot of adjusted odds ratios for 28-day mortality.

Age- and sex-adjusted single-marker logistic models are shown in **Figure 4**. For severe disease, NRR was associated with higher odds of severe HFRS (OR 2.10, 95% CI 1.70-2.66; P < 0.001). NLR (OR 1.14, 95% CI 1.07-1.23; P < 0.001), neutrophils (OR 1.16, 95% CI 1.11-1.22; P < 0.001), WBC (OR 1.08, 95% CI 1.06-1.11; P < 0.001), PCT (OR 1.06, 95% CI 1.03-1.09; P < 0.001), and creatinine were also associated with severe disease. LMR showed an inverse association (OR 0.91, 95% CI 0.82-0.99; P = 0.038), and platelets were inversely associated with severity (OR 0.99, 95% CI 0.98-0.99; P < 0.001) (**Figure 4A**).

For 28-day mortality, NRR remained associated with non-survival in the age- and sex-adjusted model (OR 1.67, 95% CI 1.31-2.19; P < 0.001) (**Figure 4B**). NLR (OR 1.08, 95% CI 1.00-1.15; P = 0.023), neutrophils (OR 1.13, 95% CI 1.07-1.20; P < 0.001), WBC (OR 1.08, 95% CI 1.04-1.12; P < 0.001), and RBC (OR 2.33, 95% CI 1.32-4.28; P = 0.004) were also associated with mortality, whereas PLR, SII, LMR, monocytes, creatinine, PCT, lymphocytes, and platelets did not show statistically significant adjusted associations in the displayed raw-unit models. In per-IQR sensitivity analyses, NRR was associated with severe disease (OR 3.17 per IQR, 95% CI 2.28-4.57; P < 0.001) and 28-day mortality (OR 2.22 per IQR, 95% CI 1.52-3.37; P < 0.001). Across the single-marker models, the maximum VIF was 1.13 and all Hosmer-Lemeshow P values were >0.05; these diagnostics did not indicate major multicollinearity or gross lack of fit, although they do not overcome the sparse mortality events.

### 3.6 Exploratory time-to-event analysis

**Figure 5:**
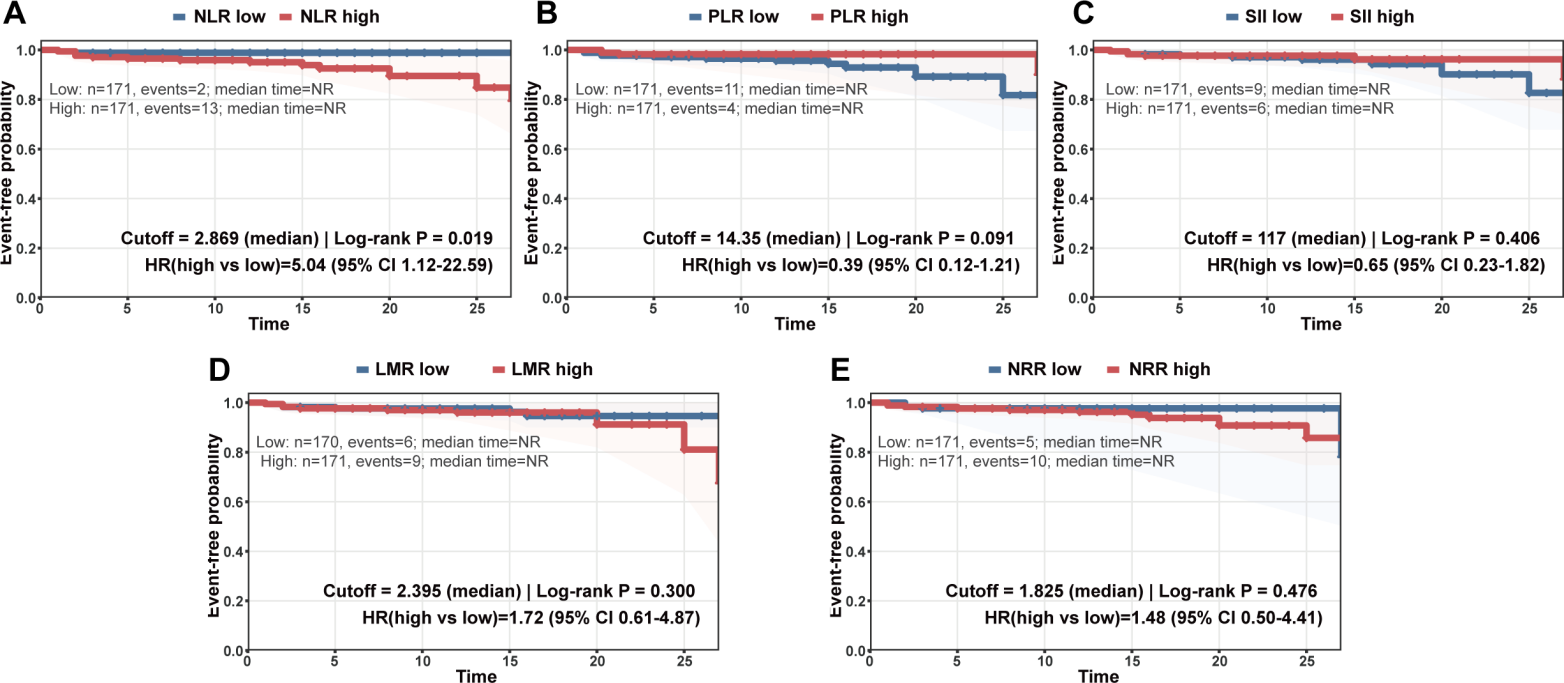
Exploratory Kaplan-Meier analyses of CBC-derived inflammatory indices for 28-day event-free survival in the derivation cohort. **(A)** NLR, cutoff 2.869 (median). **(B)** PLR, cutoff 14.35 (median). **(C)** SII, cutoff 117.0 (median). **(D)** LMR, cutoff 2.395 (median). **(E)** NRR, cutoff 1.825 (median).

Exploratory Kaplan–Meier analysis further illustrated the distinct behavior of the five indices (**Figure 5**). NLR high versus low groups showed significant separation in 28-day event-free survival (cutoff 2.869; low n = 171, events = 2; high n = 171, events = 13; HR 5.04, 95% CI 1.12-22.59; log-rank P = 0.019). In contrast, NRR did not show significant separation under the same median-based strategy (cutoff 1.825; low n = 171, events = 5; high n = 171, events = 10; HR 1.48, 95% CI 0.50-4.41; log-rank P = 0.476). PLR showed an inverse, non-significant pattern (cutoff 14.35; HR 0.39, 95% CI 0.12-1.21; log-rank P = 0.091), while SII and LMR were not significant. In the sensitivity analysis using Youden Index-derived cutoffs, stronger separation was observed for several markers, including NLR, PLR, SII, and NRR (**Supplementary Figure 1**).

### 3.7 External validation cohort results

**Figure 6:**
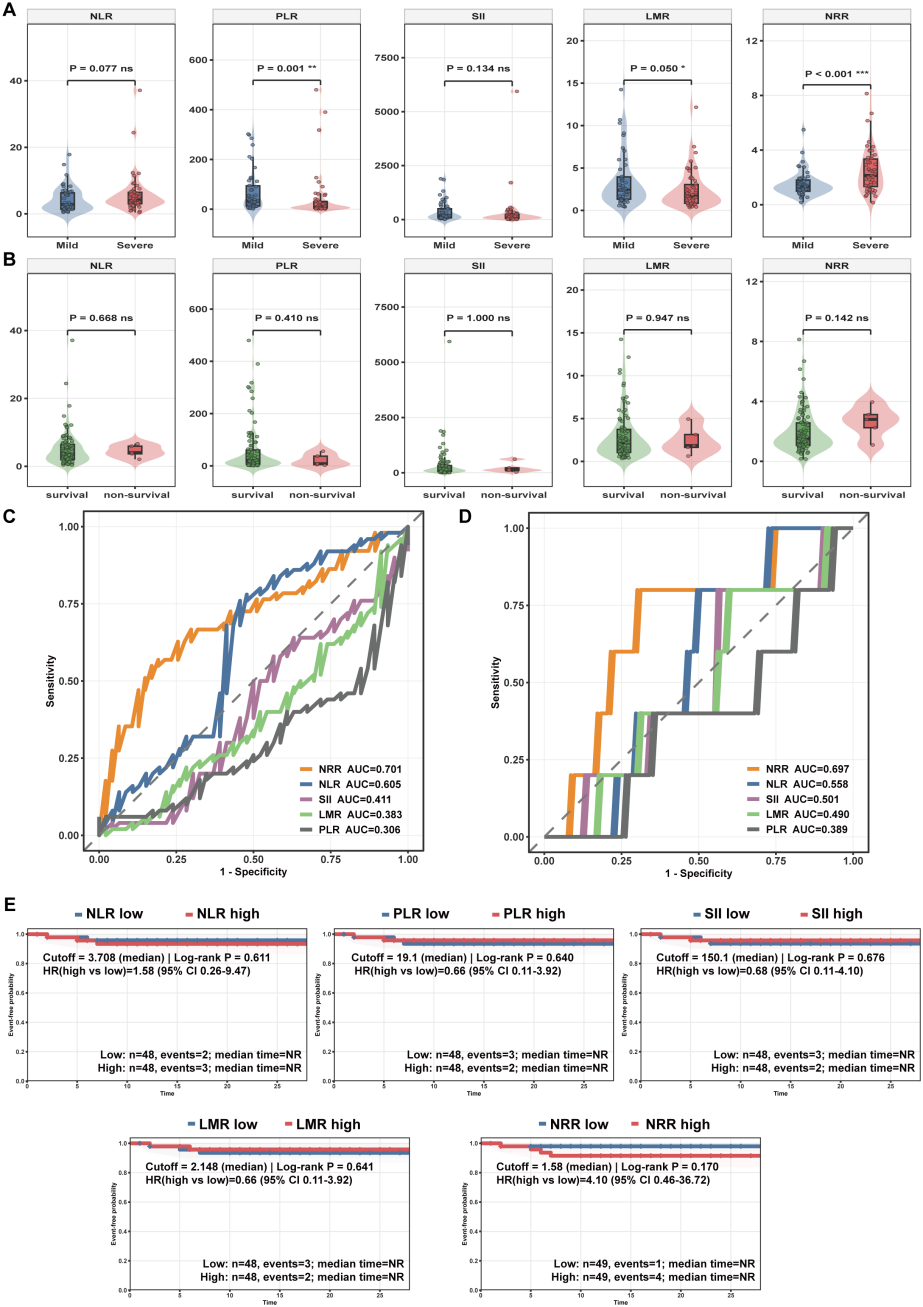
External validation **(A)** Violin/box plots comparing NLR, PLR, SII, LMR, and NRR between mild/non-severe and severe HFRS. In this figure, the “Mild” group denotes the combined mild/moderate non-severe category, and the “Severe” group denotes the combined severe/critical category. **(B)** Violin/box plots comparing the same five indices between 28-day survivors and non-survivors. P values are shown above each comparison. **(C)** ROC curves of the five CBC-derived indices for severe disease in the external validation cohort. Raw AUC values are shown in the plot legend. **(D)** ROC curves of the five CBC-derived indices for 28-day all-cause mortality in the external validation cohort. Raw AUC values are shown in the plot legend. **(E)** Exploratory Kaplan–Meier curves for 28-day event-free survival using marker-specific median cutoffs in the validation cohort. Cutoff values, log-rank P values, HRs for high versus low groups, 95% CIs, group sizes, event counts, and median survival times are displayed within each panel. *: p < 0.05, **: p < 0.01, ***: p < 0.001, ns: not significant (p > 0.05).

The external validation cohort included 100 patients, of whom 95 survived and 5 died within 28 days. Baseline characteristics according to 28-day survival status are summarized in **Table 2**. In severity analyses, NRR was significantly higher in severe than in non-severe HFRS (P < 0.001) and showed the highest AUC among the five CBC-derived indices for severe disease (AUC 0.701) (**Figure 6A**). Non-survivors showed numerically higher neutrophil counts, WBC counts, and NRR values than survivors, although these differences were not statistically significant, likely reflecting the small number of mortality events. Length of stay was significantly shorter among non-survivors (P < 0.001), consistent with early fatal events (**Figure 6B**).

**Table 2:** Baseline characteristics and early clinical course in the external validation cohort according to 28-day survival status.

Characteristic	Survivors (n=95)	Non-survivors (n=5)	P value
Cohort descriptor			
Patients, n	95	5	
Demographics and presentation			
Age, years	53.00 [35.50, 68.50]	55.00 [54.00, 68.00]	0.296
Male sex, n (%)	67 (70.5)	4 (80.0)	1.000
Routine laboratory markers and clinical course			
Neutrophils, ×10^9/L	7.04 [4.67, 11.20]	11.91 [10.95, 14.70]	0.076
Lymphocytes, ×10^9/L	2.16 [1.03, 4.25]	3.65 [1.64, 3.95]	0.347
Monocytes, ×10^9/L	0.99 [0.42, 2.48]	1.82 [0.89, 2.41]	0.453
Red blood cells, ×10^12/L	4.58 [4.14, 4.96]	4.56 [4.19, 4.79]	0.438
Platelets, ×10^9/L	44.00 [24.50, 71.00]	30.00 [20.00, 59.00]	0.574
White blood cells, ×10^9/L	12.40 [6.92, 17.86]	19.58 [13.75, 24.30]	0.141
Length of stay, days	15.00 [11.50, 20.00]	5.00 [2.00, 6.00]	<0.001
CBC-derived inflammatory indices			
NLR	3.61 [2.00, 6.45]	4.03 [3.70, 5.96]	0.662
PLR	19.58 [6.71, 60.74]	8.22 [5.06, 37.34]	0.405
SII	150.68 [65.66, 328.72]	120.82 [119.24, 218.08]	0.993
LMR	2.17 [1.05, 3.74]	1.84 [1.64, 3.14]	0.941
NRR	1.52 [1.06, 2.56]	2.80 [2.24, 3.12]	0.140

ROC analysis for severe disease showed that NRR had the highest raw AUC among the five CBC-derived indices (AUC 0.701), followed by NLR (0.605), SII (0.411), LMR (0.383), and PLR (0.306) (**Figure 6C**). The raw AUC values below 0.50 for PLR, LMR, and SII should be interpreted in relation to marker directionality. For 28-day mortality, NRR also showed the highest raw AUC among the five indices (AUC 0.697), followed by NLR (0.558), SII (0.501), LMR (0.490), and PLR (0.389) (**Figure 6D**). In age- and sex-adjusted single-marker logistic models, NRR remained associated with severe disease (OR 1.92, 95% CI 1.29–3.10; P = 0.004). However, its adjusted association with 28-day mortality was not statistically significant (OR 1.20, 95% CI 0.66-1.92; P = 0.484), and no CBC-derived index showed a significant adjusted association with mortality in the validation cohort. Median cut-off Kaplan-Meier analyses showed no significant 28-day event-free survival separation for any index, including NRR (cutoff 1.58; HR 4.10, 95% CI 0.46-36.72; log-rank P = 0.170) (**Figure 6E**). Overall, the validation cohort supported the reproducibility of the NRR signal for severe-disease stratification but did not provide definitive external validation for mortality prediction.

## 4 Discussion

In this expanded retrospective derivation cohort of adults with HFRS, CBC-derived inflammatory indices showed distinct and disease-specific behavior. NRR showed promising discrimination for severe disease and was closely comparable with PCT and absolute neutrophil count in the ROC analysis. For 28-day mortality, however, NLR and absolute neutrophil count showed the highest AUCs, while NRR showed a lower but still informative AUC and a significant age- and sex-adjusted association with non-survival. The external validation cohort partly supported these findings by confirming a reproducible severe-disease signal for NRR, but mortality validation was limited by the small number of deaths.

These patterns are biologically plausible in the context of HFRS. Neutrophils are linked to endothelial activation, barrier dysfunction, and inflammatory tissue injury during hantavirus infection (3–8). Anchoring neutrophil burden to an RBC-related denominator may partly incorporate circulatory concentration context in a disease characterized by plasma leakage and hemoconcentration (7, 8, 18). Nevertheless, RBC count is only an indirect surrogate. It may be affected by baseline anemia, hydration status, sex, altitude, bleeding, and fluid therapy, and it should not be interpreted as a direct measure of plasma leakage. Direct validation with serial hematology, fluid balance, hematocrit trajectories, and endothelial leakage markers is needed.

The comparison between NRR and absolute neutrophil count is clinically important. Absolute neutrophils remain a direct, simple, and strongly performing marker in the expanded analysis. NRR may add context by scaling neutrophil burden to RBC-related status, but the incremental value of this ratio beyond neutrophil count alone remains unproven. Future work should test whether NRR improves model performance when added to neutrophils, PCT, and organ-dysfunction scores such as SOFA. Until such analyses are externally validated, NRR should be framed as an adjunct to routine assessment rather than as a replacement for established clinical indicators.

The inverse behavior of PLR and the weak performance of SII appear to be disease-specific rather than general limitations of platelet-derived indices. Thrombocytopenia is a hallmark of HFRS and worsens with disease severity; therefore, platelet-containing ratios can decrease as clinical risk increases. This explains the raw AUC values below 0.50 for PLR and helps avoid a misleading interpretation that PLR contains no biological information. In HFRS, however, a low PLR is less intuitive as a unidirectional high-value-risk biomarker than NLR or NRR.

Under the uniform median cut-off strategy in the derivation cohort, NLR, but not NRR, showed statistically significant event-free survival separation. In the external validation cohort, none of the five median cut-off Kaplan-Meier comparisons reached statistical significance, although NRR showed the largest numerical hazard ratio with a very wide confidence interval. These findings reinforce that cutoff-based mortality stratification remains exploratory and event-limited. Youden-derived thresholds may produce stronger apparent separation, but such thresholds are outcome-optimized and prone to optimism in retrospective data; they should not be interpreted as validated clinical cutoffs.

The external validation analysis provides important information on transportability. NRR retained the highest severe-disease AUC among the five CBC-derived indices in the validation cohort (0.701) and remained associated with severe disease after age and sex adjustment. For 28-day mortality, NRR also had the highest AUC among the five CBC-derived indices in the validation cohort, but the adjusted odds ratio was not statistically significant and the Kaplan-Meier confidence interval was wide. Thus, the validation data support NRR primarily as a candidate marker for severe-disease stratification, whereas its mortality-prediction utility remains unconfirmed.

First, its retrospective design may be susceptible to residual confounding and selection bias. Although an independent external validation cohort was included, the derivation and validation cohorts were each obtained from a single institution, rather than from a multicenter network, which may limit generalizability and leave center-specific effects unaccounted for. Second, mortality events were infrequent, with 15 deaths in the derivation cohort and 5 deaths in the validation cohort, limiting model stability, precision of effect estimates, and reliability of time-to-event analyses. Third, analyses were restricted to complete cases without imputation; therefore, bias related to non-random missingness cannot be excluded. Fourth, serial trajectories of CBC-derived indices were not evaluated, and direct measurements of plasma leakage or hemoconcentration were unavailable, limiting mechanistic interpretation of NRR. These findings should therefore be confirmed in prospective multicenter studies before routine clinical application.

Despite these limitations, the updated analysis provides a more conservative and transparent assessment of CBC-derived indices in HFRS. NRR appears most useful for severe-disease stratification, and this signal was reproduced in the external validation cohort. NLR remains particularly informative for short-term mortality in the derivation cohort, whereas mortality validation for all CBC-derived indices was constrained by the very small number of validation deaths. NRR and NLR may therefore provide complementary admission information, pending prospective confirmation.

## 5 Conclusion

In this expanded retrospective derivation cohort with an independent 100-patient external validation cohort, NRR showed promising and externally reproducible performance for identifying severe HFRS. NRR was also associated with 28-day mortality in the derivation cohort, but this mortality association was not statistically confirmed in the validation cohort. NRR may reflect a combined neutrophil-related inflammatory and RBC-related circulatory context, but this interpretation remains indirect. NRR should be considered a candidate adjunctive marker rather than a replacement for absolute neutrophil count, PCT, or organ-dysfunction assessment, and prospective multicenter studies are required before routine clinical implementation.

## Data Availability

The raw data supporting the conclusions of this article will be made available by the authors, without undue reservation.

## Supplementary material

Supplementary Figure 1 Exploratory Kaplan–Meier sensitivity analyses using Youden index-derived cutoffs in the derivation cohort. Kaplan–Meier curves show 28-day event-free survival according to low versus high groups for the five admission CBC-derived inflammatory indices: **(A)** NLR, cutoff 2.641; **(B)** PLR, cutoff 6.094; **(C)** SII, cutoff 74.29; **(D)** LMR, cutoff 2.819; and **(E)** NRR, cutoff 4.415. Event-free survival was defined as survival without all-cause death within 28 days after admission. Group sizes, event numbers, median survival times, log-rank P values, and hazard ratios for high versus low groups with 95% confidence intervals are displayed within each panel.
