# Supplementary figures and images for: Admission complete blood count-derived inflammatory indices for risk stratification in hemorrhagic fever with renal syndrome: comparative performance of NLR, PLR, SII, LMR, and the neutrophil-to-red blood cell ratio
Source: Front Immunol. 2026 Jun 30;17:1863714. doi: 10.3389/fimmu.2026.1863714 (PMC13365174; doi:10.3389/fimmu.2026.1863714)

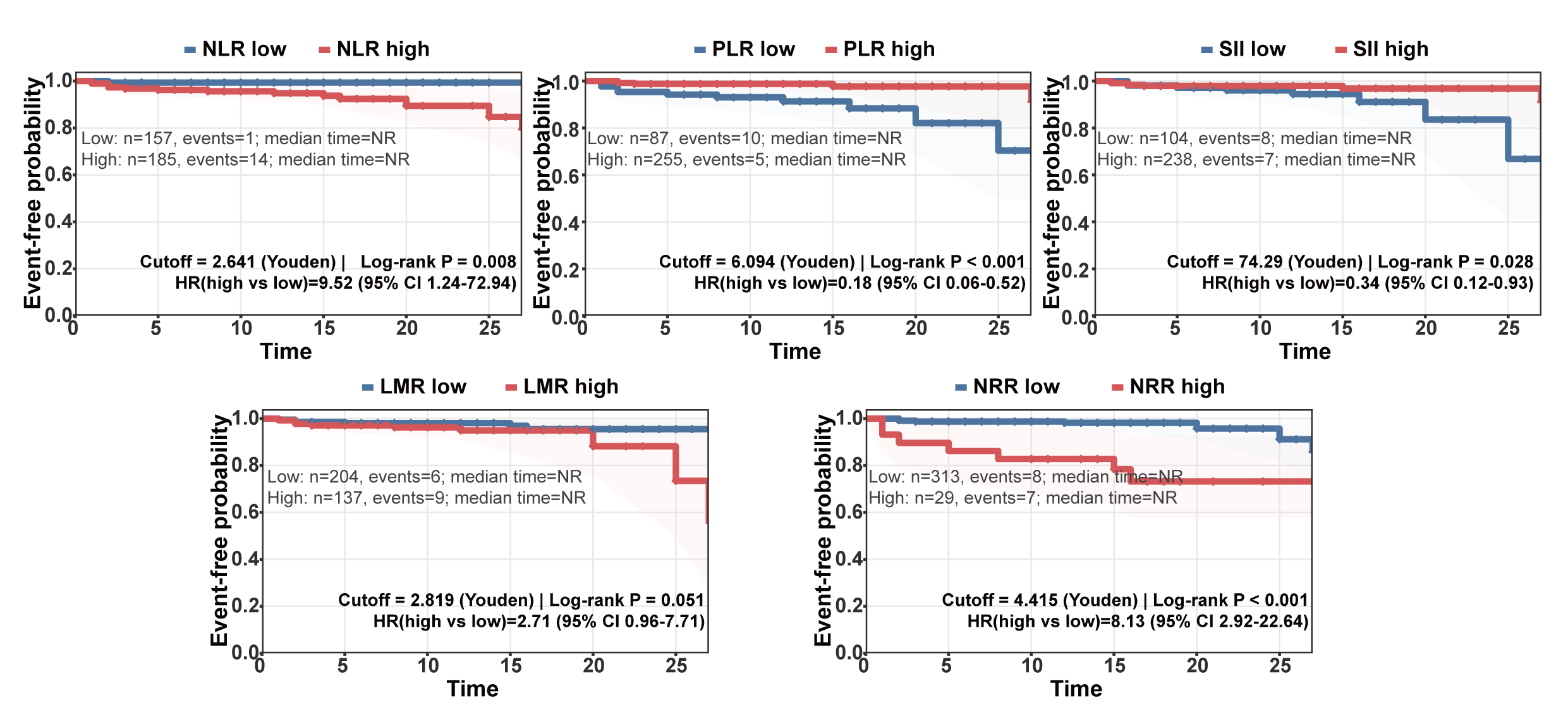

Supplement: Supplementary Figure 1 — Exploratory Kaplan–Meier sensitivity analyses using Youden index-derived cutoffs in the derivation cohort. Kaplan–Meier curves show 28-day event-free survival according to low versus high groups for the five admission CBC-derived inflammatory indices: (A) NLR, cutoff 2.641; (B) PLR, cutoff 6.094; (C) SII, cutoff 74.29; (D) LMR, cutoff 2.819; and (E) NRR, cutoff 4.415. Event-free survival was defined as survival without all-cause death within 28 days after admission. Group sizes, event numbers, median survival times, log-rank P values, and hazard ratios for high versus low groups with 95% confidence intervals are displayed within each panel. [file Image1.tif]
